# New Insights in the Contribution of Voltage-Gated Na_v_ Channels to Rat Aorta Contraction

**DOI:** 10.1371/journal.pone.0007360

**Published:** 2009-10-07

**Authors:** Aurélie Fort, Magali Cordaillat, Catherine Thollon, Guillermo Salazar, Ilana Mechaly, Nicole Villeneuve, Jean-Paul Vilaine, Sylvain Richard, Anne Virsolvy

**Affiliations:** 1 Inserm U637, Université Montpellier1 & 2, Montpellier, France; 2 Cardiovascular Division, Institut de Recherches Servier, Suresnes, France; 3 Inserm U583, Université Montpellier2, Montpellier, France; Yale School of Medicine, United States of America

## Abstract

**Background:**

Despite increasing evidence for the presence of voltage-gated Na^+^ channels (Na_v_) isoforms and measurements of Na_v_ channel currents with the patch-clamp technique in arterial myocytes, no information is available to date as to whether or not Na_v_ channels play a functional role in arteries. The aim of the present work was to look for a physiological role of Na_v_ channels in the control of rat aortic contraction.

**Methodology/Principal Findings:**

Na_v_ channels were detected in the aortic media by Western blot analysis and double immunofluorescence labeling for Na_v_ channels and smooth muscle α-actin using specific antibodies. In parallel, using real time RT-PCR, we identified three Na_v_ transcripts: Na_v_1.2, Na_v_1.3, and Na_v_1.5. Only the Na_v_1.2 isoform was found in the intact media and in freshly isolated myocytes excluding contamination by other cell types. Using the specific Na_v_ channel agonist veratridine and antagonist tetrodotoxin (TTX), we unmasked a contribution of these channels in the response to the depolarizing agent KCl on rat aortic isometric tension recorded from endothelium-denuded aortic rings. Experimental conditions excluded a contribution of Na_v_ channels from the perivascular sympathetic nerve terminals. Addition of low concentrations of KCl (2–10 mM), which induced moderate membrane depolarization (e.g., from −55.9±1.4 mV to −45.9±1.2 mV at 10 mmol/L as measured with microelectrodes), triggered a contraction potentiated by veratridine (100 µM) and blocked by TTX (1 µM). KB-R7943, an inhibitor of the reverse mode of the Na^+^/Ca^2+^ exchanger, mimicked the effect of TTX and had no additive effect in presence of TTX.

**Conclusions/Significance:**

These results define a new role for Na_v_ channels in arterial physiology, and suggest that the TTX-sensitive Na_v_1.2 isoform, together with the Na^+^/Ca^2+^ exchanger, contributes to the contractile response of aortic myocytes at physiological range of membrane depolarization.

## Introduction

Over the last two decades, there has been increasing evidence for the presence of tetrodotoxin-sensitive (TTX_S_) voltage-gated Na^+^ currents (I_Na_) in arterial smooth muscle cells (SMCs). Most of these observations have been made in primary cultured cells derived from large human and mammalian arteries [1]–[5]. I_Na_ have also been recorded in freshly isolated vascular myocytes, although the process of enzymatic dissociation is critical, and is a potential limiting factor in recording these currents [6]. Voltage-gated Na^+^ channels (Na_v_) are usually responsible for the initiation and propagation of the action potential in excitable cells including, typically, neurons, skeletal muscle and cardiac cells. However, they could play a different role in arteries. It has been shown that some Na_v_ channels regulate intracellular Ca^2+^ ([Ca^2+^]_i_) in human coronary myocytes in primary culture. This regulation is effective at baseline and involves the tonic control of Ca^2+^ influx [7]. Yet, even now, the functional role of Na_v_ channels in intact arteries is unknown.

At the molecular level, Na_v_ channels are composed of a membrane-spanning pore-forming α subunit (∼260 kDa), which may be associated with different auxiliary β-subunits [8]. Nine genes encoding functional Na_v_ channel α-subunits (named Na_v_1.1 through Na_v_1.9) have been cloned, electrophysiologically characterized, and exhibit the appropriate ion permeation, voltage sensing and inactivation properties. Four β-subunits, named β1, β2, β3 and β4 have also been cloned and shown to regulate Na_v_ channel α-subunit gating and expression levels [8]. Most Na_v_ channels isoforms can be classified according to their sensitivity to the specific Na_v_ channel blocker TTX. TTX-sensitive (TTX_S_) isoforms are inhibited at nanomolar concentrations. They are preferentially expressed in the nervous system (Na_v_1.1, Na_v_1.2, Na_v_1.3, Na_v_1.6, and Na_v_1.7) or in adult skeletal muscle (Na_v_1.4). TTX-resistant (TTX_R_) isoforms (inhibited in the micromolar range) are the cardiac isoform (Na_v_1.5) and isoforms expressed in the peripheral nervous system (Na_v_1.8 and Na_v_1.9). Various gene products coding for Na_v_ channels have been detected in arterial myocytes [5], [9].

Despite an increasing number of studies providing evidence for the presence of I_Na_ in arterial cells, no information is available as to whether or not Na_v_ channels play a functional role in arteries. In the present work, we looked for a physiological role of Na_v_ channel activity in the control of aortic contraction in the rat. Our results show that the brain-type Na_v_1.2 channel is, surprisingly, expressed in the muscular layer of the aorta, and is likely to contribute to contraction.

## Materials and Methods

### Isolation of arteries and myocytes

The investigation conformed to the guidelines for the Care and Use of Laboratory Animals (NIH, N°.85–23, revised 1996) and European directives (96/609/EEC) and was approved by the *Comité Régional d'Ethique sur l'expérimentation animale* of *Languedoc-Roussillon* on the Use and Care of Animals. Male Sprague-Dawley rats (22–25 weeks) were anesthetized with an intraperitoneal injection of pentobarbital (150 mg/kg). The thoracic aorta was removed, immersed in a physiological saline solution (PSS) cleaned and processed for the different studies. The thoracic aorta was removed, immersed in a physiological saline solution (PSS) cleaned and processed for the different studies. Myocytes were isolated by enzymatic dissociation using collagenase type I (1 mg/ml) and elastase (0.5 mg/ml, Sigma) as described before [10]. The composition of the PSS was as follows (in mmol/L): 140 NaCL, 5 KCl, 1 MgCl_2_, 0.5 KH_2_PO_4_, 0.5 Na_2_HPO_4_, 2.5 CaCl_2_, 10 HEPES, 10 glucose, pH 7.4.

### Western blotting

Total protein was extracted from whole tissues (brain and aorta) and the presence of Na_v_ channel proteins was determined by Western blot analysis using a rabbit polyclonal anti-Pan-Na_v_ channel antibody (Alomone; 1∶500). A rabbit polyclonal GAPDH antibody (FL:335, Santa Cruz) was used to determine the cellular fraction of the crude protein extract. Tissues were homogenized in ice-cold buffer containing 20 mmol/L Tris-HCl, 150 mmol/L NaCl, 10 mmol/L dithiotreitol and protease inhibitor cocktail (Roche). Proteins (10 µg for brain and 50 µg for aorta) were separated on a 4.5% SDS-polyacrylamide gel and transferred to nitrocellulose membranes. Antibody specificity was validated by incubating with the peptide antigen (1 µg peptide for 1 µg antibody). After washing in Tris-buffered saline containing 0.1% Tween-20 (TBS-T), membranes were blocked in TBS-T containing 5% skimmed milk powder and incubated with the anti-Pan Na_v_ channel antibody or polyclonal GAPDH antibody. After washing in TBS-T, membranes were incubated with a horseradish peroxidase-conjugated anti-rabbit secondary antibody (1∶1000). Bound immunoglobulins were revealed with SuperSignal West Pico chemiluminescent substrate (Pierce) and signal recorded using a Kodak image station. The rabbit polyclonal GAPDH antibody (FL:335, Santa Cruz) was used to estimate the cellular fraction of the crude protein extract on the same blots.

### RNA extraction, end-point RT-PCR and real-time PCR

Total RNA was extracted from rat aorta or from isolated cells using Tri reagent (Sigma) according to manufacturer's instructions. DNase-treated (DNase I, Invitrogen) total RNA (1–2 µg) was transcribed into cDNA using Superscript II reverse transcriptase (Invitrogen) and random primer oligonucleotides (Invitrogen). Gene-specific primers for rat Na_v_ channel isoforms, α-actin and GAPDH were designed based on sequences available through PubMed (for sequences see Table 1). End-point PCR reactions were run on 50–100 ng cDNA using a HotStartTaq® polymerase (Qiagen). Amplification was carried out as follows: an initial activation step at 95°C for 15 min, followed by 35 cycles of 30 s at 95°C, 1 min at 59°C and 1 min at 72°C, with final extension for 8 min at 72°C. PCR products were visualized under UV light after electrophoresis on 2% agarose gels containing ethidium bromide. Negative controls were run without reverse transcriptase. Each primer pair was validated by amplification of total RNA from positive controls (rat brain, heart and skeletal muscle) and direct sequencing of the PCR product (Genome Express). Real-time quantitative PCR was performed in a Light Cycler System (Roche) in combination with the Absolute QPCR SYBR Green Capillary mix (Abgene). After a hot start (15 min at 95°C), the parameters for amplification were: 1 s at 95°C, 5 s at 60°C and 10 s at 72°C for 45 cycles. Primers selected in table 1 were of equal efficiency (E_ff_ = 1.9) across the range of template concentrations (1–10 ng cDNA). Expression levels normalized with GAPDH were calculated relative to the less abundant isoform (Na_v_1.6) using the E_ff_^−ΔΔCt^ method.

**Table 1:** Nucleotide sequences of the specific primers used to detect Na_v_ channel α and β isoforms by RT-PCR.

Isoform	Accession number	Forward primer	Reverse primer	Product size (pb)
Nav 1.1	X03368	CATCATCTTCGGCTCGTTCT	GCTTGTCACATAATCGCTCTGG	316
Nav 1.2	X03639	TCTTCGGCTCATTCTTCACC	GTTGGTCATCTCCTGACTCTGGT	311
Nav 1.3	Y00766	CGGCTCAAAGAAACCTCAGA	TCGAGAGAATCACCACCACA	305
Nav 1.4	NM_013178	TGGGAATAGCAGTGATGCTG	AAGGAGCCCAGGAAGATGAT	228
Nav 1.5	NM_013125	GAGAACCCAGACCACGGTTA	GCCTCGGTGTTCCTTCTTGAG	305
Nav 1.6	L39018	GTTCATCGGTGTCATCATCG	CAAGGCAAACATTTTGAGCA	355
Nav 1.7	AF000368	TTCGGCTCATTCTTCACGTT	CACTCCCCAGTGAACAGGAT	359
Nav 1.8	NM_017247	CACGGATGACAACAGGTCAC	GATCCCGTCAGGAAATGAGA	151
Nav 1.9	NM_019265	GCTCCTTGAGCAGACCAAC	TTTATGCACAGCCACTGAGG	222
Nav β1	NM_017288	GTGTATGGGATGACCTTCAAA	GTAGTCGCCAGAGTGG	267
Nav β2	NM_012877	GATGCCTGGCTACCTCGCCCT	AACCTGAAGCTGGAGCGGTTT	276
Nav β3	NM_139097	GACTCTGGCCTCTACAC	GCGTCTGACTACCTTGC	260
Nav β4	NM_001008880	ATAACAGCGAAACATCCAGG	CACGAAGCAAGTGTATCTGC	188
β actin	NM_031144	TACCCCATTGAACACGGC	TGGGCACAGTGTGGGTGAC	289
GAPDH	NM_0170008	AGAACATCATCCCTGCATCC	TCCACCACCCTGTTGCTGTA	367

Each set of primers was designed from mRNA sequences with the Genbank accession numbers indicated above, using the primer design software Light Cycler Probe Design (Roche). They generated PCR products of the predicted length in base pairs.

### Immunohistochemistry

Double immunofluorescence labeling for Na_v_ channels and smooth muscle α-actin was carried out on cryostat sections of rat aorta embedded in OCT (Tissue Tek®), and immersed in liquid N_2_. After fixation in ethanol/acetone (v/v), tissue sections were first blocked with 5% normal goat serum (Sigma) in PBS containing 1% BSA, then incubated with the first primary antibody, washed with PBS and incubated with the first fluorescent secondary antibody. Sections were then blocked with 5% normal horse serum (Sigma), incubated with the second primary antibody and finally incubated with the second fluorescent secondary antibody. Nuclei were counterstained with 5 µmol/L TOTO®-3 iodide (Molecular Probes). Sections were coverslipped with fluorescent mounting medium (DakoCytomation) and visualized using a BioRad MRC-1024 laser scanning confocal imaging system (Montpellier RIO imaging platform). The primary antibodies used were: anti-Pan Na_v_ channel antibody for the first labeling step, and a mouse monoclonal anti-smooth muscle α-actin antibody (clone 1A4, Sigma; 1/1000) for the second. Secondary antibodies were, respectively, Texas Red®-conjugated goat anti-rabbit IgG and fluorescein-conjugated horse anti-mouse IgG, both diluted 1/100 (Vector Laboratories). Negative controls followed the same protocol but without primary antibody, or with the inclusion of peptide antigens (1 µg of peptide for 1 µg of antibody).

### Vascular reactivity

Experiments were performed as previously described [11]. The aorta was cut into 2–3 mm-wide rings. The endothelium was removed by rubbing and the rings were placed in conventional organ bath chambers filled with PSS, maintained at 37°C and continuously bubbled with O_2_. Changes in isometric tension were recorded using an IT1-25 force transducer and an IOX computerized system (EMKA Technologies, Paris, France). After a 60 min equilibration period at a resting tension of 2 g (optimal resting tension), the viability and contractility of each arterial segment was assessed with 1 µmol/L phenylephrine (PE). Acetylcholine causes endothelium-dependent vasorelaxation. Thus the absence of functional endothelium was confirmed on each aortic ring by the lack of response to acetylcholine (1 µmol/L) on top of PE effect. Concentration-response curves were generated by a cumulative increase in the concentration of various vasoconstrictors. The blockade of α_1_-adrenergic receptors was carried out by a 10 min pre-incubation period with 10 µmol/L prazosin. Its efficacy, controlled with 1 µmol/L phenylephrine or with 1 mmol/L tyramine, was maintained even after several wash-outs. Specific protocols are detailed in the legends.

### Membrane potential

Rings of thoracic aorta (2–3 mm long) were cut open along the longitudinal axis and pinned down to the bottom of an experimental chamber, endothelial side upward. All aorta segments were superfused continuously at 5 mL/min with oxygenated PSS (37°C, pH 7.4, bubbled with O_2_). The membrane potential was measured in subintimal vascular smooth muscle cells, after crossing both the endothelial cell layer and the internal elastic laminae, using glass microelectrodes (30–40 MΩ) filled with 3M KCl [12]. Recordings were made using a conventional high-impedance amplifier (V 180, Biologic). The membrane potential was monitored on a digital storage oscilloscope (2211, Tektronix). Signals were digitized and analyzed using specific software (EMKA Technologies). After a resting period, aorta preparations were exposed to increasing KCl concentrations, from 5 to 15 mmol/L, during stable recording from one smooth muscle cell. Changes in membrane potential were compared to time-matched recordings performed with PSS. For some experiments, a wash-out step was carried out after exposure to increased K^+^ solutions.

### Data analysis

All data are expressed as mean ± standard error of the mean (s.e.m.), for *n* experiments. Data were statistically analyzed using either the Student's t test for unpaired or paired values depending on experimental procedures, or a two-way ANOVA followed by a Bonferroni post-test, as specified in the legends. P values lower than 0.05 were considered significant.

## Results

### Identification of Na_v_ channel isoforms in the rat aorta

**Figure 1:**
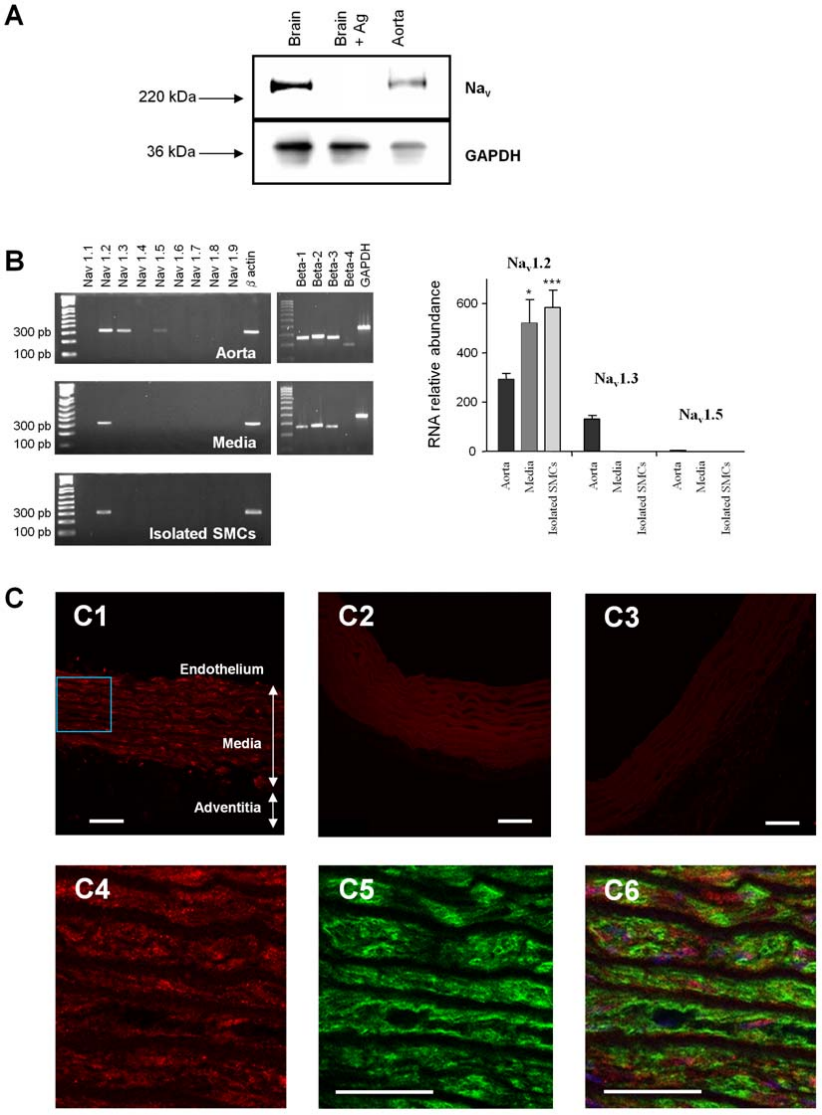
Identification of Na_v_ channels in the rat aorta and isolated vascular myocytes. (A) Western blot analysis of protein extracts from control tissues (brain; 10 µg of total protein extract per tissue) and aorta (50 µg of total protein extract per tissue) performed using an anti-Pan Na_v_ channel antibody. Labeling specificity was assessed in the brain by pre-incubating the primary antibody with the peptide antigen. Cellular protein content was estimated from the GAPDH signal (lower panel). Arrows indicate the migration of the molecular weight marker (220 and 36 kDa). (B) Analysis of Na_v_ transcripts in total RNA extracted from rat aorta, aortic media and freshly isolated SMCs: representative images obtained after end-point RT-PCR. β-actin or GAPDH primers were used as positive controls to validate reverse transcription. The panel at right shows a sample quantification of Na_v_ isoform transcripts. The expression of Na_v_1.2, 1.3 and 1.5 was evaluated by quantitative real-time RT-PCR. Transcript levels were normalized to that of the GAPDH housekeeping gene in each sample and compared in the whole aorta, media layer and freshly isolated SMCs. Data are expressed as means ±s.e.m. of five experiments, each performed in triplicate. *p<0.05; ***p<0.001, unpaired *t-test*. (C) Immunolocalization of Na_v_ channels in aortic tissue. The upper panel represents typical confocal images obtained from successive sections of rat aorta using a 20X objective; (C1) Specific anti-Pan labeling; arrows indicate the endothelium, media and adventitial layers; (C2) negative control without primary antibody; (C3) negative control with the peptide antigen. Lower panels correspond to high magnification (60X objective) images of the inset in C1, showing immunofluorescence staining for Na_v_ channels (C4), α-actin (C5), and double immunofluorescence staining with TOTO counterstaining (blue) (C6). *Scale bars* - 50 µm.

Western blotting with a specific Na_v_ channel α-subunit antibody (anti-Pan) showed the presence of Na_v_ channel proteins in the rat aorta (Fig. 1A). A positive control from brain tissue revealed a band at around 250 kDa. Antibody specificity was validated in the brain, known to express Na_v_ channels highly, by incubating with the peptide antigen which abolished the signal. The observation of similar bands in aortic protein extracts indicated the presence of Na_v_ channels. We determined the molecular identity of these Na_v_ channels by RT-PCR, using primers specific for each known isoform. Each primer pair yielded a single product with the predicted length and sequence (data not shown) in control tissues (brain, heart and skeletal muscle). In the whole rat aorta, three transcripts were detected: Na_v_1.2, Na_v_1.3 and Na_v_1.5 (Fig. 1B). Only Na_v_1.2 was detected in the media and in freshly isolated aortic myocytes. The four β−isoform transcripts were also detected, although the β_4_ isoform was not detected in the media (Fig. 1B).

### Localization of Na_v_ channels

Aortic tissue sections were double-labeled with an anti-Pan antibody (red fluorescence) and a smooth muscle α-actin antibody (green fluorescence) to examine the localization of Na_v_ channels (Fig. 1C). Red fluorescence was observed in the media (Fig. 1C1). Labeling specificity was validated using negative controls without primary antibody (Fig. 1C2) or with the peptide antigen (Fig. 1C3). Under high magnification, Na_v_ channels were found to be homogenously distributed throughout the media (Fig. 1C4 ) in cells identified as vascular SMCs by green labeling for smooth muscle α-actin (Fig. 1C5 and Fig. 1C6).

### Effect of veratridine on aortic contraction

**Figure 2:**
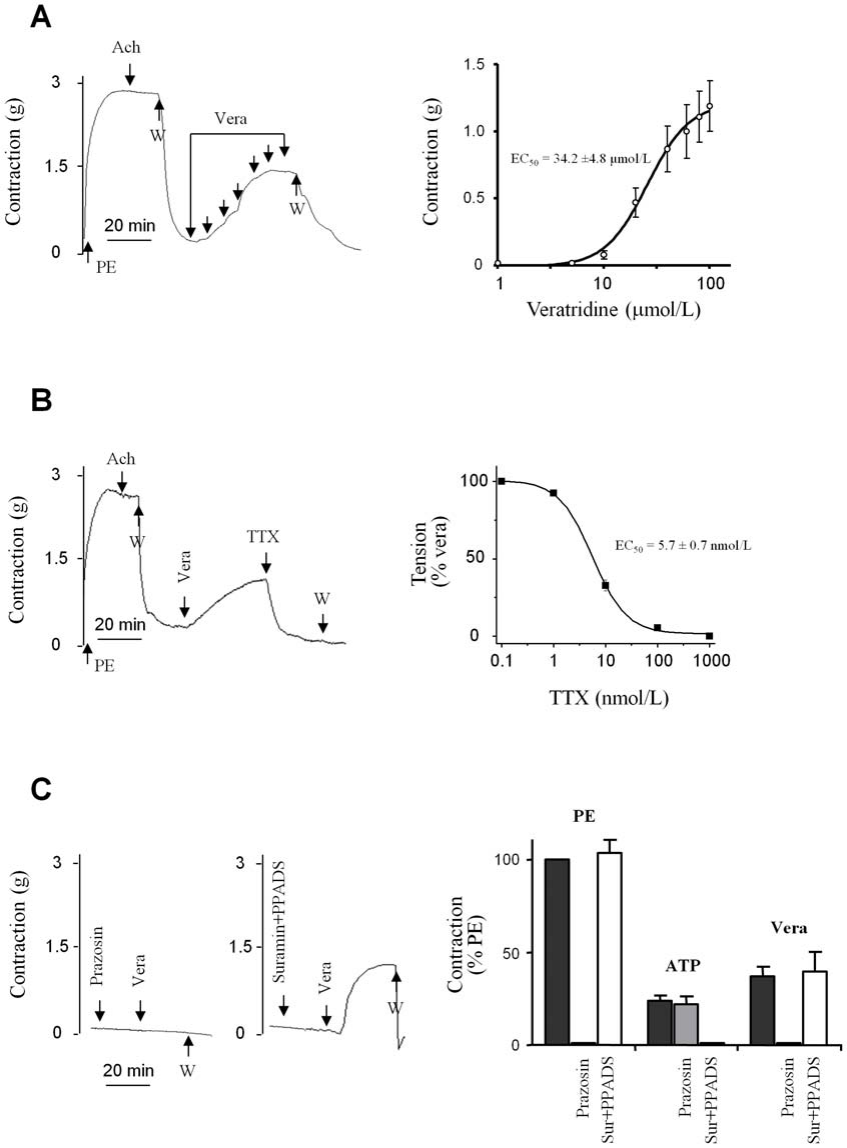
Contractile response of rat aortic rings to veratridine. (A) Veratridine-induced vasoconstriction. *Left panel*: typical recordings of the isometric response to cumulative doses of veratridine (vera). The absence of endothelium was first confirmed by the lack of vasorelaxing effect of 1 µmol/L acetylcholine (Ach) on the contraction evoked by a sub maximal concentration of phenylephrine (PE; 1 µmol/L). After washing (W), increasing concentrations of veratridine (arrows), from 5 to 100 µmol/L, were added. *Right panel*: concentration-response curves of veratridine (EC_50_ = 34.2±4.8 µmol/M). (B) Vasorelaxing effect of TTX (1 µmol/L) on the contraction induced by 100 µmol/L veratridine (representative isometric response, *left panel*). The dose-response curve was obtained by addition of cumulative concentrations of TTX (0.1 nmol/L to 1 µmol/L) after maximal contraction was evoked by 100 µmol/L veratridine. Values are expressed as a percentage of the maximal contraction induced by veratridine. (C) Effect of veratridine in the presence of prazosin or suramin plus PPADS (*left panel*). The graph (*right panel*) represents the contractions induced by PE (1 µmol/L), ATP (100 µmol/L) and veratridine (100 µmol/L) under basal conditions or in the presence of either 10 µmol/L prazosin or a cocktail of suramin (300 µmol/L) and PPADS (30 µmol/L). Data are expressed as a percentage of the response induced by PE (1 µmol/L). All values represent means ±s.e.m. of five experiments, each performed in triplicate.

The vasoconstrictors PE, arginin-vasopressin (AVP) and endothelin-1 (ET-1) as well as the depolarizing agent KCl induced a dose-dependent contraction of endothelium-denuded rat aortic rings with similar maximal amplitudes at saturating concentrations (PE: 3.8±0.4 g; AVP: 3.3±0.3 g; ET-1: 4.0±0.6 g; KCl: 3.7±0.9 g). The EC_50_ values and mean increases in tension (Table 2) were consistent with previously published data [13]–[15]. Subsequently, Na_v_ channel activity was studied using veratridine. Veratridine is an alkaloid with agonist properties which binds to site 2 of Na_v_ channels and causes a dramatic slowing of their inactivation and deactivation, leading to sustained Na^+^ influx [2], [16], [17]. As shown in Figure 2A, veratridine induced a concentration-dependent increase in tension with an EC_50_ value of 34.2±4.8 µmol/L. At saturating concentrations, veratridine induced an increase in tension of 1.2±0.2 g, corresponding to 30% of the maximal contraction induced by the vasoconstrictor). The subsequent addition of TTX inhibited the veratridine-induced contraction in a dose-dependent manner with an EC_50_ value of 5.7±0.7 nmol/L (Fig. 2B). Complete recovery was observed after the wash-out of veratridine, and TTX had no effect on the resting tension (data not shown).

**Table 2:** Characteristics of the contractile responses of endothelium-denuded aortic rings to various agonists.

Agonist	EC_50_	Emax (g)
KCl	8.5±1.1 mmol/L	3.70±0.93
PE	120±30 nmol/L	3.75±0.43
AVP	11.2±2.3 nmol/L	3.34±0.34
ET-1	4.4±1.5 nmol/L	4.00±0.62

The agonists used are KCl, phenylephrine (PE), arginine-vasopressin (AVP), and endothelin-1 (ET-1). Results are presented as E_max_, corresponding to the maximal effect elicited by each agonist expressed in terms of isometric tension (g), and EC_50_ values (in mmol/L or nmol/L), indicating the concentration at which 50% of the maximal effect is induced. Values represent the mean±s.e.m. of 5 animals, with experiments performed in duplicate.

To assess any possible contribution of sympathetic neurotransmitters release (norepinephrine and ATP) to the effect of veratridine, we performed experiments in the presence of prazosin, an antagonist of the α_1_-adrenergic receptors responsible for the vasoconstrictive action of norepinephrine, and/or of a cocktail of selective and competitive antagonists of ATP-stimulated responses [18]. A saturating concentration of prazosin (10 µmol/L) fully antagonized the potentiating effects of a maximally effective concentration of the α_1_-adrenergic receptor agonist PE (1 µmol/L; Fig. 2C), and of tyramine (100 µmol/L; data not shown), an indirect sympathomimetic agent which triggers transmitter release from adrenergic terminals and prolongs their action [19]–[21]. While prazosin (10 µmol/L) had no effect *per se*, it abolished the contraction induced by veratridine. A cocktail composed of the P2Y receptor antagonist suramin (300 µmol/L) and the P2X receptor antagonist pyridoxal-phosphate-6-azophenyl-2′, 4′-disulphonic acid (PPADS; 30 µmol/L), which had no effect *per se*, abolished the vasoconstrictive effect of ATP (100 µmol/L). This cocktail had no effect on the contraction induced by veratridine (Fig. 2C).

### Na_v_ channel activity unmasked by low KCl

The experiments detailed above show that the activation of α_1_-adrenergic receptors could be involved in the effect of veratridine on aortic contraction, presumably via neuronal Na_v_ channel activity and catecholamine release. In order to specifically investigate the functional activity of Na_v_ channels at the aortic myocytes level, subsequent experiments were performed in the presence of prazosin (which we have shown to be necessary and sufficient to abolish the effect of veratridine) and, eventually, of the suramin/PPADS cocktail. We also reasoned that the use of massive concentrations of KCl, resulting in large depolarizations, could directly activate Ca^2+^ channels. To specifically target Na_v_ channels activity and avoid the direct recruitment of L-type Ca^2+^ channels, depolarization was induced by the addition of low concentrations of KCl.

**Figure 3:**
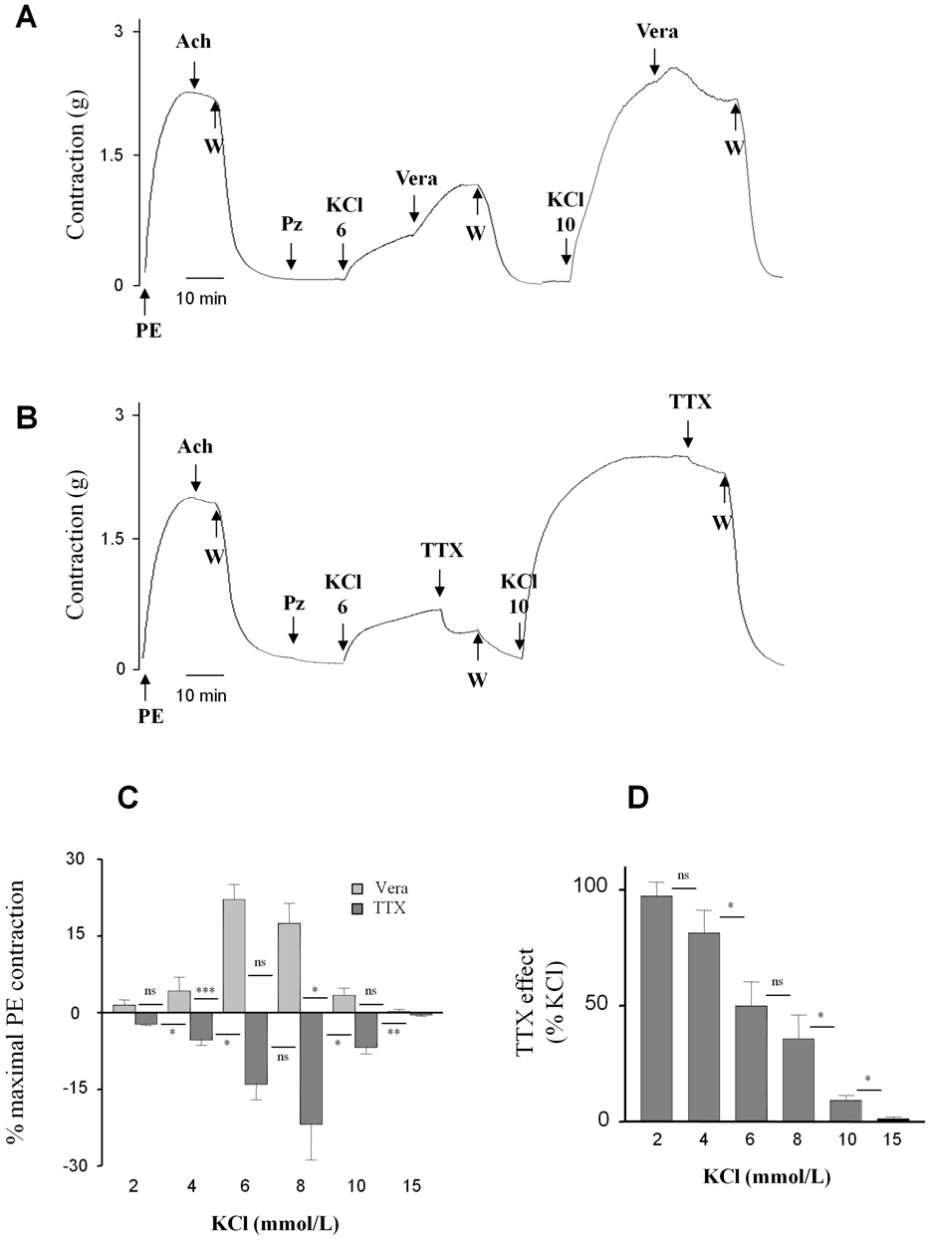
Dependence on KCl of contractile response to veratridine and TTX in the presence of prazosin. The absence of endothelium was confirmed as described above. Representative recordings of isometric responses to veratridine (vera; 100 µmol/L) (A) or tetrodotoxin (TTX; 1 µmol/L) (B) after contraction induction by KCl (6 and 10 mmol/L) in the presence of 10 µmol/L prazosin (Pz). (C) Summary of results of the experiments described in A and B: the effects of veratridine (100 µmol/L) or TTX (1 µmol/L) on the contraction induced by KCl were expressed as a percentage of maximal contraction induced by PE (1 µmol/L). (D) Effects of TTX expressed as a percentage of KCl-induced contraction. Values represent the mean±s.e.m. of 6 animals, with experiments performed in duplicate. *p<0.05; **p<0.01; ***p<0.001, paired *t-test*.

Figure 3A illustrates that, in the presence of prazosin, veratridine (100 µmol/L) potentiates the contraction induced by the addition of 6 mmol/L KCl to the extracellular bath. A similar but smaller effect was seen after the addition of 10 mmol/L KCl. The amplitude of the effect of veratridine depended in fact on the concentration of KCl and followed a bell-curve between 2 and 15 mmol/L KCl. The maximal response was obtained at 6–8 mmol/L KCl (Fig. 3C) and represented 20% of the maximal contraction induced by 1 µmol/l PE. Veratridine had no apparent effect at KCl concentrations below 2 mmol/L or above 15 mmol/L.

**Figure 4:**
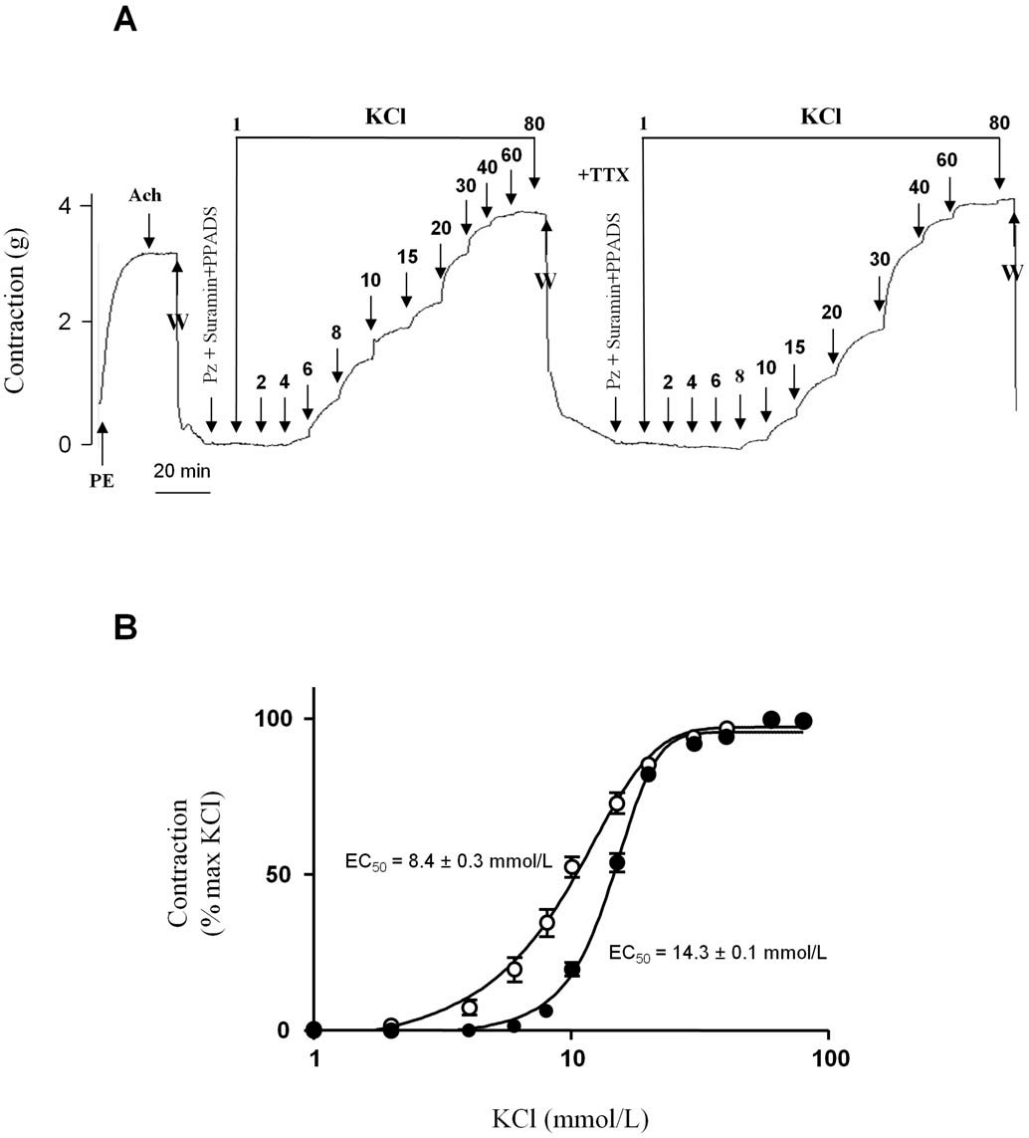
Involvement of aortic SMC Na_v_ channels in the contractile response to KCl. The absence of endothelium was confirmed as described above. After washing and 15 min of incubation with prazosin (Pz; 10 µmol/L), suramin (300 µmol/L) and PPADS (30 µmol/L), increasing concentrations of KCl (1 to 100 mmol/L) were added in the absence or presence of TTX (1 µmol/L). (A) Representative recording of the isometric response. (B) Dose-responses to KCl in the absence (▒) or in the presence of 1 µmol/L TTX (•). Data are expressed as a percentage of the maximal KCl-induced contraction and were analyzed with a non-linear fit function to determine EC_50_ values. Values represent means±s.e.m. of 5 animals, with experiments performed in quadruplicate.

Figure 3(B and C) show that TTX (1 µmol/L) inhibited the contraction induced by the addition of low concentrations of KCl to the bath whereas no effect was observed with concentrations of KCl higher than 15 mmol/L. Thus, the vasorelaxing effects of TTX mirrored the enhancement induced by veratridine between 2 and 15 mmol/L KCl, with maximal inhibition observed at 6–8 mmol/L, once again corresponding to 20% of the maximal contractile response (Fig. 3C). Interestingly, these data, expressed as a percentage of KCl-induced contraction (Fig. 3D), reveal that the contribution of the TTX-sensitive component of contraction to the KCl response decreases monotonically from 100 % at 2 mmol/L to 0 % at 15 mmol/L. These data suggest that most of the vasoconstrictive response observed at the lowest KCl concentrations is mediated by Na_v_ channels. This contribution was confirmed by comparing the response to increasing concentrations of KCl in the presence or absence of TTX (1 µmol/L) (Fig. 4). TTX prevented the contraction induced by KCl at concentrations below 8 mmol/L whereas contraction was significantly attenuated at concentrations between 8 and 15 mmol/L (Fig. 4). The EC_50_ value for KCl-induced contraction increased from 8.4±0.3 mmol/L to 14.3±0.1 mmol/L in the presence of TTX (Fig. 4B). When TTX was used to block Na_v_ channels, the KCl-induced depolarization triggered contraction via the direct recruitment of Ca^2+^ channels.

### The Na^+^-Ca^2+^ exchanger links Na_v_ channels activity and contraction

**Figure 5:**
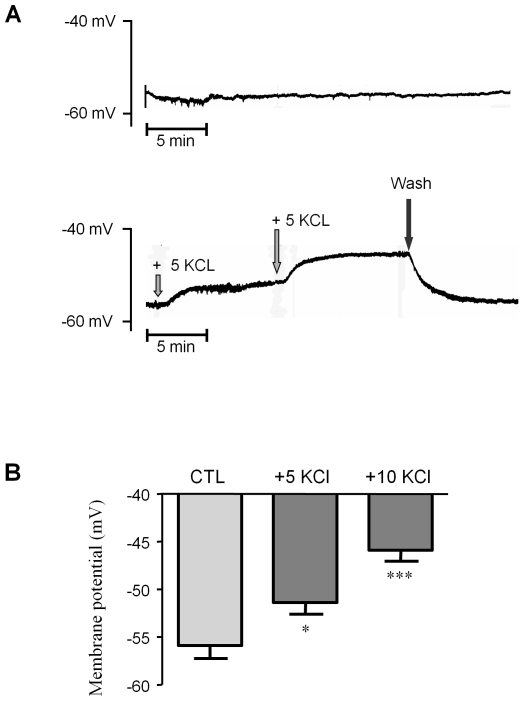
Membrane potential determination. Membrane potentials were recorded in the SMCs of aortic rings under basal conditions and after addition of low KCl concentrations, using glass microelectrodes. (A) Representative recordings illustrating membrane potential stability in control experiments (upper panel) and depolarizations induced by the cumulative addition of 5 mmol/L KCl (lower panel). (B) Averaged membrane potentials under basal conditions (CTL) and after addition of 5 mmol/L and 10 mmol/L KCl (as described). *p<0.05; ***p<0.001; two-way ANOVA followed by a Bonferroni post-hoc analysis. Values represent means ±s.e.m. of 4 animals, with experiments performed in duplicate.

**Figure 6:**
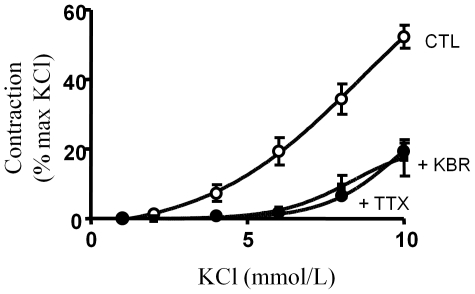
Na^+^-Ca^2+^ exchanger activity mediates the aortic contractile response after addition of low KCl concentrations. Dose-response curves for KCl-induced aortic contraction in physiological saline solution (CTL;▒), and in the presence of either 1 µmol/L TTX (•) or 1 µmol/L KB-R7943 (▪). Data were obtained by the cumulative addition of KCl (1 to 10 mmol/L) to aortic rings pre-incubated with the prazosin (10 µmol/L), suramin (300 µmol/L) and PPADS (30 µmol/L) cocktail.

We next measured the membrane potential of vascular SMCs in aortic preparations, and its variation after the addition of low concentrations of KCl to the extracellular bath [12]. The average RMP recorded in basal buffer (which contains 5.0 mmol/L KCL) as a reference, was −55.9±1.4 mV (n = 8). Figure 5A illustrates the depolarizing effect of low concentrations of KCl, in comparison with stable time-matched recordings performed in control solution. The addition of 5 mmol/L and 10 mmol/L KCl to the bath depolarized the RMP to −51.4±1.2 mV (n = 8) and −45.9±1.2 mV (n = 8) respectively (Fig. 5B). These values were too negative to open the L-Type Ca^2+^ channel which has a threshold above −40 mV at physiological concentration of Ca^2+^ [22]. To explain the link between Na_v_ channel activation and vascular contraction, we tested the participation of the Na^+^-Ca^2+^ exchanger by using the blocker KB-R7943 reported to inhibit the reverse mode of exchange (Ca^2+^ influx/Na^+^ efflux) [23]. Figure 6 shows that KB-R7943 (1 µmol/L) blocked the contraction induced by low concentrations of KCl, thereby reproducing the inhibitory effects of TTX. In addition, KB-R7943 had no additive effect in presence of TTX (data not shown). Thus, the Na^+^ influx mediated by Na^+^ channels activates the reverse mode of the Na^+^-Ca^2+^ exchanger, which promotes Ca^2+^ entry and subsequent contraction.

## Discussion

Voltage-gated I_Na_ have been recorded in vascular myocytes [1]–[5]. However, the physiological role of Na_v_ channels *in vivo* has remained an open question. Although Na_v_ channels in peripheral vascular nerve endings contribute to arterial contraction, our study defines a TTX_S_ Na_v_ channel isoform (Na_v_1.2) expressed in myocytes of the rat aortic media and provides the first evidence of its contribution to aortic contraction at a physiological range of depolarization.

### Na_v_ channels in rat arteries

We have shown the presence of Na_v_ channels in the rat aorta. We identified three mRNA transcripts: two neuronal TTX_S_ isoforms (Na_v_ 1.2 and Na_v_ 1.3), and the cardiac TTX_R_ isoform Na_v_1.5. Significantly, only the Na_v_1.2 isoform was present in the rat aortic SMCs. Taken together, these findings are consistent with previous reports establishing the presence of Na_v_ isoforms in human and animal arterial SMCs [9], [24]. Na_v_ channels were distributed in the membranes, as expected for voltage-gated proteins, and in the cytoplasm of the rat aortic SMCs. Similar cytoplasmic localization has been described before, for example, in the soma of primary vestibular neurons and in macrophages, in relation with Na_v_ channel storage or/and trafficking to the membrane [25], [26].

### Na^+^ channel-dependent contraction originating in the myocytes

One key finding of our study is evidence of a marked dependence of the contraction of rat aortas upon Na_v_ channel activity. The Na_v_ channel agonist veratridine triggered a dose-dependent aortic contraction with an EC_50_ value of 34.2 µM, consistent with previous observations [27], [28]. This response reflects a genuine effect on TTX-sensitive Na_v_ channels, as proven by its specific inhibition by TTX (EC_50_ = 5.7±0.7 nmol/L). Its suppression by the α_1_-adrenergic receptor antagonist prazosin suggested that part of this response is mediated through activation of Na_v_ channels in sympathetic perivascular nerve-terminals. Subsequent induction of catecholamines release is known to regulate arterial blood pressure via α_1_-adrenergic receptors in the rat aorta [29]–[31]. In line with this view, catecholamines are involved in the intense pressor effect induced by the scorpion toxins TsTX-V and TsTX-1, isolated from *Tityus serrulatus* venom, that act on Na_v_ channels [32].

We defined experimental conditions under which a Na_v_ channel-dependent contraction likely to originate in the SMCs was unmasked. In absence of α_1_-adrenergic and ATP receptors stimulation, addition of low concentrations of KCl (2–10 mM) triggered a contraction potentiated by veratridine (100 µM) and blocked by TTX (1 µM). It attained up to 20% of the maximal contraction induced by potent agonists such as PE, AVP, ET-1 and KCl, and accounted for most of the contraction evoked by low KCl depolarization (Fig. 3D). It has probably been overlooked in the past, most likely due to the narrow range of KCl concentrations required (2–15 mmol/L) to evidence it and the lack of TTX use, essential to its identification. At concentrations higher than 15 mmol/L, KCl-induced depolarization is expected to promote the direct opening of voltage-gated Ca^2+^ channels, thereby bypassing Na_v_ channels. The lack of an effect of TTX on the aortic resting tension, together with the fact that membrane depolarization induced by the addition of low concentrations of KCl (2–15 mmol/L) is required to unmask this component of contraction, reveals that Na_v_ channels are closed at physiological RMPs. Our measurement of the RMP (−56 mV) is consistent with other reports [33], [34] and with the estimated activation threshold of the Na_v_1.2 isoform; that is between −60 mV and −50 mV [35], [36]. Interestingly, the window current determined by the overlap of the steady-state activation and inactivation curves of the Na_v_1.2 isoform is expected to generate a persistent influx of Na^+^ between the activation threshold (between −60 mV and −50 mV) and −40 mV [35], [36]. The membrane potentials set by the addition of low concentrations of external KCl (5 mmol/L: −51 mV, and 10 mmol/L: −46 mV) are consistent with the activation of Na_v_ channels in this range of potentials, which thereby enables a sustained window Na^+^ influx. These potentials are too negative to account for the activation of rat aortic-SMC L-type Ca^2+^ channels that have a threshold of activation above −40 mV at physiological concentration of Ca^2+^ [22]. The blocking effect of KB-R7943 is in favor of the involvement of the Na^+^/Ca^2+^ exchanger working in reverse mode, which provides the route for Ca^2+^ entry (against Na^+^ extrusion) and activation of contraction. This interpretation is consistent with our previous report of a Na^+^/Ca^2+^-mediated TTX-sensitive Ca^2+^ influx in cultured human coronary myocytes [7].

### Functional significance

Our results taken as a whole - immunochemistry, RT-PCR and functional studies - suggest that Na_v_1.2 channels in rat aortic myocytes provide a mechanism by which slight depolarization controls arterial contraction. These Na_v_ channels are likely to translate small changes in the RMP into a fine-tuning of Ca^2+^ entry, thereby inducing moderate contraction consistent with physiological regulation. The activation of aortic SMC Na_v_ channels occurs within a narrow window of potentials, thus providing cells with an effective mechanism for the maintenance of elevated [Na^+^]_i_, the induction of Na^+^/Ca^2+^ exchanger activity, and consequently, the entry of Ca^2+^ independently of Ca^2+^ channels activation. Although the participation of the Na^+^/Ca^2+^ exchanger in vascular SMCs contraction has been described, there is a lack of information regarding the mechanisms leading to its activation by intracellular Na^+^. Our discovery of the control of aortic contraction by Na_v_ channels and its sensitivity to KB-R7943 provides an excellent candidate for such a mechanism. Vascular Na_v_ channels may thus be involved in critical pathophysiological situations and contribute to the role of the Na^+^/Ca^2+^ exchanger in vasoconstriction [37]–[39].

**In summary**, our study provides the first evidence of a TTX-sensitive component of tension activated by KCl-induced depolarization in the aorta, with the unexpected conclusion that the brain-type Na_v_1.2 isoform is involved in this process. The Na^+^-dependent activation of the reverse mode of the Na^+^/Ca^2+^ exchanger provides the necessary link to the influx of Ca^2+^ and the subsequent contraction of myocytes within the physiological range of membrane depolarizations. The description of a TTX-sensitive contraction originating in vascular myocytes expressing the Na_v_1.2 isoform constitutes a major and novel discovery.
